# The world is getting smaller: The importance of a global approach to general practice research and practice

**DOI:** 10.1080/13814788.2022.2143686

**Published:** 2022-11-23

**Authors:** Christian Mallen

**Affiliations:** Faculty of Health, Keele University, Keele, UK

Not even Nostradamus predicted what has happened over the past two years. The world often seemed to stand still as everyone watched with shock and fear as the events related to the COVID-19 pandemic unfolded. The pandemic has opened the eyes of patients, clinicians, and policymakers to the importance of global health. It is increasingly apparent that health problems in one part of the world can rapidly impact our populations. There are many examples of how health in local populations have been influenced by global patterns of disease. A mutation of the monkeypox virus results in previously unheard-of cases across Europe, the war in Ukraine triggers unprecedented increases in the costs of daily living and widening social inequality, and the devastating effects related to global warming continue to have an ever-increasing impact on health. Health is truly international and can no longer be contained in regional silos. The world is getting smaller every day.

General practitioners increasingly need to think globally. Our academic endeavours are typically focussed on regional and national clinical priorities, yet the skills possessed can benefit much broader international populations as demonstrated throughout the COVID-19 pandemic. This expertise can be translated to tackling other global challenges – but this does represent a shift in how we currently work. The clinical and academic workforce needs to prepare our existing and future practitioners for the new challenges they will inevitably face during their careers. GPs need to plan, prepare and be ready for the next problem encountered – whatever that may be and wherever it may come from.

Primary care and general practice are critical to improving health and well-being in low- and middle-income countries. The World Health Organisation’s Sustainable Development Goal 3 aims to achieve ‘good health and wellbeing for all’ [1]. This is an ambitious target that can only be met by primary care. Primary care can help reduce health inequalities, providing the broadest range of services in a cost-effective manner embedded in local communities [2]. Primary care takes a holistic approach to the patient journey, considering the wider needs of individuals and their families [3]. Growing the evidence base to support this, especially in low- and middle-income countries is a key component to future success.

Internationally we have seen significant investment in global health research through organisations including the Bill and Melinda Gates Foundation and the National Institute for Health Research [4,5]. Such funding is aimed at tackling the most pressing global challenges and whilst as clinicians we are often impatient for results, we are already starting to see major advances from working more closely together. Novel technologies used to develop the first COVID-19 vaccines are now directed towards other serious infections, such as Ebola [6]. For the first time, effective malaria vaccination feels like a real possibility and the research community are calling for multimorbidity in low and middle-income countries to be actively targeted [7].

Many general practice academics feel that primary care is often the poor relation to secondary care research. That may be true but increasingly, policymakers and funders are aware that to make major inroads to improving health and wellbeing internationally, primary care must be firmly at the centre of the picture.

The *European Journal of General Practice* is committed to publishing the best research from primary care worldwide, especially when it influences policy and practice. Whilst most of our readers may be based in Europe, we are keen to learn lessons from clinicians and academics across the world. We have all learnt from each other during the COVID-19 pandemic, taking best practice from one country to support our own patients [8]. General practice is always stronger when we work together, and when we learn from our shared experiences.
